# Is Resting State Functional MRI Effective Connectivity in Movement Disorders Helpful? A Focused Review Across Lifespan and Disease

**DOI:** 10.3389/fneur.2022.847834

**Published:** 2022-04-14

**Authors:** Bethany L. Sussman, Sarah N. Wyckoff, Jennifer Heim, Angus A. Wilfong, P. David Adelson, Michael C. Kruer, Maria Jose Gonzalez, Varina L. Boerwinkle

**Affiliations:** ^1^Division of Neuroscience, Barrow Neurological Institute at Phoenix Children's Hospital, Phoenix, AZ, United States; ^2^Department of Research, Phoenix Children's Hospital, Phoenix, AZ, United States; ^3^Division of Pediatric Neurology, Barrow Neurological Institute at Phoenix Children's Hospital, Phoenix, AZ, United States; ^4^Division of Pediatric Neurosurgery, Barrow Neurological Institute at Phoenix Children's Hospital, Phoenix, AZ, United States; ^5^Departments of Child Health, Neurology, Genetics and Cellular & Molecular Medicine, University of Arizona College of Medicine – Phoenix, Phoenix, AZ, United States; ^6^Departments of Pediatrics, SBH Health System, Bronx, NY, United States

**Keywords:** resting state functional MRI, effective connectivity, dystonia, movement disorders, subcortical, basal ganglia

## Abstract

In the evolving modern era of neuromodulation for movement disorders in adults and children, much progress has been made recently characterizing the human motor network (MN) with potentially important treatment implications. Herein is a focused review of relevant resting state fMRI functional and effective connectivity of the human motor network across the lifespan in health and disease. The goal is to examine how the transition from functional connectivity to dynamic effective connectivity may be especially informative of network-targeted movement disorder therapies, with hopeful implications for children.

## Introduction

The brain motor networks to supports movement are comprised of complex circuits of cortical and subcortical locations. A common clinical application goal of functional neuroimaging has been to develop the ability to make inferences about phenotype and treatment. Since movement disorders are ultimately network disorders, functional neuroimaging investigations of motor networks and movement benefit from being grounded in the context of network activity (i.e., connectivity). Resting state fMRI, in particular, is attractive to consider due to the relative ease and low patient/subject demand during scans. A question then is in what context is rs-fMRI of potential use.

This review is divided into two main sections with sub-sections. The first section “Functional Connectivity” briefly introduces the concepts of structural and functional connectivity as well as the neural systems that support them in the context of movement. We then go on to provide a focused review of resting state fMRI functional connectivity in the context of healthy adults, development/aging, movement disorders, and treatment. In the second major section “Advancements through Effective Connectivity,” we briefly introduce concepts and methods of effective connectivity and give a focused review of effective connectivity findings related to healthy adults and movement disorders and the potential gains from using effective connectivity methods. In order to appreciate the context of the topics discussed in this review, it is additionally important to understand it in the context that flexible motor behavior is characterized by the ability to attain the same task outcomes using different movement plans, often in a way that adapts to the environment (1).

## Functional Connectivity

### Motor Network Functional Connectivity in Healthy Adults

Studies of motor network (MN) resting state fMRI (rs-fMRI) functional connectivity (FC) build upon those from structural connectivity (SC) in healthy adults. SC refers to anatomical connections and FC refers to correlated activity. While SC often implies and is thought to support FC, the absence of a known anatomical (structural) connection does not negate the possibility of a functional connection (most commonly measured as correlated activity). This is, in part, due to the way that SC and FC are differently determined (physical connection vs. correlated activity) and, in part, because FC can be indirect or the structural connections supporting it may be unknown. Together, studies of SC and FC show that flexible motor behavior is generated and tightly regulated by sophisticated physiological networks residing in the cortex and subcortical structures through a series of topographically organized parallel loops, known as the direct, indirect, and hyperdirect pathways (2–7). The direct pathway is thought to initiate movement (8, 9), the indirect pathway and the hyperdirect pathway both are involved in movement suppression (5, 8, 9).

Although these network loops are configured in parallel, as evidenced by neuron tracing studies in primates and non-primate animal models and corroborated in diffusion tensor imaging (DTI) tractography in healthy adults, they are not completely independent (4, 7, 8, 10). These pathways use several common structures to support movement. These circuits are classically organized as follows: In the **direct pathway (simplified)**, the striatum (consisting of the caudate nucleus and the putamen) receives excitatory cortical input, the putamen, which handles a significant amount of motor input to the striatum, sends inhibitory signal to the globus pallidus internus (GPi) and substantia nigra pars reticulata (SNr), which, in turn, *disinhibits* the thalamus. Since the thalamus is generally excitatory toward cortex, this causes excitation toward cortex that supports movement (9). In contrast, in the **indirect pathway**, motor cortex sends excitatory signal to the striatum, which then inhibits the external segment of the globus pallidus (GPe) causing a disinhibition of the subthalamic nucleus (STN). In turn, the STN sends excitatory signal the GPi/SNr causing the GPi/SNr to inhibit the thalamus ultimately stopping the thalamus from sending excitatory signal to cortex (9). As such, the “start and stop” supported by these two pathways both initiates and ends movements as well as supports smooth, coordinated movement when functioning together appropriately. Dysfunctions in one or both of these pathways can lead to non-volitional and jerky movements and/or difficulty initiating movements (9, 11, 12). The final (known), more recently discovered pathway, the **hyperdirect pathway**, also ultimately inhibits the thalamus (and suppresses movement), though more quickly (5). It does so by bypassing the striatum and GPe with an excitatory projection directly to the STN which then follows the same progression as in the indirect pathway, ultimately inhibiting (or rather, not exciting) movement (5). In addition to the hyperdirect pathway, understanding of cortical-subcortical motor loops has also been expanded to incorporate connections with the cerebellum (13, 14) [see Quartarone et al. (10) and Milardi et al. (15) for review].

Such parallel yet interdependent networks yield means to compensate for local network pathology. Evidence for the compensation-yielding loops is shown through isolated lesions of pallidum and striatum in adults, which are not always associated with severe motor dysfunction (10, 16). This suggests that, although these pathways appear pivotal to movement, there are likely mechanisms within them that compensate for a degree of perturbation or irregularity. Thus, the inherently static measures of SC and FC have provided additional insight and support of prior modalities', such as neuron tracings, findings of MN configuration and function.

#### Motor Network Functional Connectivity and Age

For the purpose of this review, further mentions of functional connectivity (FC) refers to resting state functional connectivity unless otherwise specified. FC has revealed *age*-dependent basal ganglia (BG) and related motor phenotype differences (3, 17–19). In a study of healthy 7–18-year-olds the used independent component analysis (ICA), age was positively correlated with FC within multiple BG network components including: right postcentral cortex (primary somatosensory cortex) extending to right precuneus, left caudate and thalami, and the left olfactory region extending to left putamen and amygdala (19). In the same study, in the default mode network component, FC in the right pallidum and putamen were positively correlated with age (19). Additionally, in 18–49 year-olds, FC between BG regions (e.g., between caudate head and body, between caudate body and putamen, between posterior putamen and pallidum) increased with age (3). Finally, in a study of 12–71 year-olds, FC of BG was positively correlated with age (17). In contrast, in a study of 60–82 year-olds, FC within bilateral caudate and putamen was negatively correlated with age (18). Together, this may suggest that BG FC primarily increases from age seven to middle adulthood and then decreases in the healthy aging elderly (17–19).

In terms of connections from BG to other regions, a study in adults ages 18–49 showed that FC of both the putamen and pallidum to areas of the default mode network (specifically ventromedial prefrontal cortex/posterior cingulate cortex) increased linearly with age while BG connectivity to regions in the “task-positive” (specifically somatomotor cortex) network decreased linearly with age (3). Thus, in this study, changes in FC from early to mid-adulthood are more varied between the BG and cortical regions (e.g., increased connectivity to regions such as ventromedial prefrontal cortex but decreased connectivity to somatomotor cortex) than between subcortical motor regions (primarily increased FC with age), indicating the subcortical and cortical motor structures have different age-dependent connectivity trajectories in adulthood (3). Overall, motor network FC across development does have age-related changes with primarily increased FC within deep gray structures with age (3, 17, 19), until old age, at which time it begins to decrease (18) and more variable changes in FC between deep gray and cortical networks through young to middle adulthood (3).

#### Motor Network Functional Connectivity and Motor Skill

In the healthy, MN FC is associated with motor skill and training. A recent study of trained young adult dancers showed that, compared to non-dancers, dancers had greater short-range functional connectivity density [FCD; a voxelwise data-driven metric that identifies local (short-range) and global (long-range) correlations; (20)] in bilateral sensorimotor cortex and increased long-range FCDs in bilateral putamen and right superior occipital gyrus (21). Dancers also had increased FC between the putamen and middle cingulate cortex (MCC) and *enhanced functional integration*, meaning increased correlational FC, of cortico-basal ganglia loops via the MCC. Specifically, this meant that when seeded FC was used (correlations from one specified region), the left putamen, right putamen, and left postcentral gyrus all had increased FC to MCC in dancers (21). Additionally, FCD in the sensorimotor cortex was positively correlated with the average time spent training in a week (21). This suggests that FC in these regions is related to the amount of time spent using motor programs and regions, which is relevant for clinical conditions that may affect those regions. A study investigating FC in musicians with and without musician's dystonia found that, FC within in the right putamen was increased overall in musicians with musician's dystonia compared to musicians without musician's dystonia (hyperconnectivity), and that this difference was greatest in the right anterior putamen (22). However, increased FC within the anterior right putamen was correlated with piano skill playing level in healthy controls (HC), but not those with musician's dystonia (22). Together, the results of these two studies suggest that the FC of the putamen is particularly important when it comes to motor skill building and that while increased putaminal FC supports skill building, “too much” (hyperconnectivity) may be detrimental. Thus, MN FC is associated with and is modulated by MN-utilization skills and this appears to be distinguishable from disease-related FC.

In contrast with the MN FC increases associated with motor skill training of specific complex skills, in one study of healthy aging adults (60–82 years old) no relationship between BG FC and motor skill was detected (18). Since this study was only in aging adults, it's not clear if this is the effect of age, the covariates used (e.g., Pegboard tasks in the aging study (18), as opposed to trained skill level, time spent training, disease, etc.), or a combination thereof. It is interesting to note that, in both an aging sample (18) and a sample with BG affecting disease (dystonia) (22), the degree of FC did not correlate with the respective motor skill measures for each study. Given the overall difference in the different connectivity patterns (i.e., overall, those with musician's dystonia showed increased FC in the right anterior putamen but aging was associated with decreased bilateral putaminal FC), this suggests that aging and onset of acquired disease (in this case, dystonia) may not have the same relationship with motor network FC changes and may be at two ends of the connectivity spectrum. However, it is important to note that methodological differences (e.g., ROI selection, analysis method) in these studies could have contributed to findings.

### Motor Network Functional Connectivity in Clinical Populations

#### Neurodegenerative Disease

MN FC demonstrates disease differentiating and severity attributes. Compared to healthy aging controls (HC), patients with Parkinson's disease, a neurodegenerative disease characterized by nigro-striatal dopaminergic function loss (23), show reduced FC between striatum and the thalamus, midbrain, pons, and cerebellum, and a relative absence of anti-correlations between striatum and cortical regions including sensorimotor, visual and subgenual prefrontal areas (24). Parkinson's disease FC has also shown additional broader range reduced connectivity in the supplementary motor area (SMA), left dorsolateral prefrontal cortex (DLPFC), and left putamen, and increased FC in the left primary motor cortex (M1), parietal cortex, and cerebellum compared to HC (25). Further, in Parkinson's disease, midbrain-cortical FC is associated with freezing of gait (26). These changes and associations appear largely specific to motor-related regions. This is important for potential clinical applications because this suggests FC is both sensitive to neurodegeneration and specifically spatially localizing instead of being associated with more generalized differences.

While the above studies demonstrate FC differences associated with a neurodegenerative disease with prominent motor symptoms (amongst other symptomology including some cognitive impairments) compared to typical aging, it is not clear from these studies if the FC differences seen are more specific to Parkinson's disease or are general to neurodegenerative disease. One way to investigate disease specificity is to compare to another disease with neurodegenerative pathology, such as Alzheimer's disease. While Parkinson's disease and Alzheimer's disease are both neurodegenerative, they have largely differentiated neurodegenerative patterns across the brain. Neurodegeneration in Alzheimer's disease is primarily cortical with some subcortical neurodegeneration (27) while Parkinson's disease neurodegeneration is largely within BG (23). As such, they provide an informative contrast when investigating functional brain networks. A recent meta-analysis and systematic review of FC in Alzheimer's disease found consistent differences (reduced FC) compared to typical aging in the default mode network, salience network and, to an extent, limbic networks (28). The authors note, however, that many studies restrict their investigation to specific networks (e.g., default mode), which calls into question whether differences in the DMN are truly larger than in other networks (28).

If FC is sensitive to and localizes disease process neurodegeneration, direct comparisons of atypical FC between Parkinson's and Alzheimer's patients should show, at least, spatial differences. This was demonstrated in a recent study comparing FC of BG in patients with Parkinson's disease, Alzheimer's disease, and healthy controls (HC) (29). In this study, patients with Parkinson's disease specifically showed decreased BG FC (hypoconnectivity), compared to HC and Alzheimer's disease (29). Additionally, while another study showed that Parkinson's and Alzheimer's patients both had reduced striatal FC with other regions compared to controls, reduced FC between middle striatum and was negatively correlated with different cognitive deficit's in Parkinson's (executive functions) and Alzheimer's (memory performance) disease (30). Furthermore, Parkinson's, but not Alzheimer's patients with mild cognitive impairment had decreased FC between dorsal striatum and primary sensorimotor and visual regions (30). Taken together, FC differences have been noted between Parkinson's and Alzheimer's patients when within-region FC is investigated (29) and when between-region FC is investigated (30). Thus, Alzheimer's and Parkinson's disease, while both neurodegenerative diseases, are differentiable by BG FC changes (29, 30).

#### Brain Insult (Early Life)

Differences in MN FC are not limited to neurodegenerative motor conditions. Perinatal brain insults associated with motor deficits have shown FC correlates. Neonatal prematurity showed association between thalamus-motor and BG-motor network connectivity and behavioral motor outcome scores (31). In a systematic review, neonatal diaschisis resulting in later outcomes of severe childhood motor impairments were associated with MN FC (32). Furthermore, a recent study demonstrated that the presence and typicality of FC BG networks (identified from independent component analysis; ICA) in neonates during the acute period of brain injury predicted clinically noted developmental delay and motor-tone outcomes within 6 months (33). Finally, severity of motor deficits (chronic spastic quadriplegia) after anoxic brain injury from drowning have also been correlated with the “health” of motor rs-fMRI ICA networks in children and that this can be independent of cognitive function and cognitive networks (34). Together, studies of FC after brain injury, particularly anoxic brain injury, imply that motor deficits and movement disorders can be correlated with MN FC after brain insult.

#### Acquired Movement/Motor Disorders

FC differences have also been noted in patients with acquired movement disorders, such as focal dystonias. Dystonia is a movement disorder that is characterized by involuntary muscle contractions that lead to odd postures, repetitive movements, or both (35, 36). Focal dystonia refers to dystonias that are restricted to one part of the body (35). Examples include cervical dystonia [characterized by involuntary contractions of neck muscles, causing the head to turn to one side or tilt forward or backward; (37)] and blepharospasm [characterized by involuntary eye movements such as blinking and twitching; (38)]. FC has demonstrated differences between patients with focal dystonia and HC and that these differences can be specific to the type of focal dystonia. In cervical dystonia, patients were shown to have decreased FC between the primary and secondary sensory cortices compared to controls, but increased FC within the BG, between the BG and thalamus, and between the somatosensory and motor cortices (39). In contrast, patients with blepharospasm showed only widespread reduced FC, both regional (between neighboring cortical or cerebellar regions, but not within deep gray structures) and long-range (40).

Some acquired dystonias are task specific, meaning that they almost solely occur during a specific activity (35). Task specific dystonias are thought to be, in part, due to repetitive movement associated with an activity (35). For example, writer's cramp is a focal dystonia that involves the dominant hand (and sometimes arm) and musician's dystonia generally involves the hands or the mouth (embouchure dystonia) (35). Right-handed patients with writers' cramp show FC decreases in the left primary somatosensory area as well as between the left superior parietal lobule and left dorsal premotor hand area and FC increases in left putamen (41, 42). As stated above, FC within the right anterior putamen is increased in patients with musician's dystonia compared to HC (22). Additionally, patients with spasmodic dysphonia have been shown to have a mixture of increased and decreased FC between deep gray, cerebellar, and cortical regions thought to subserve language and articulation compared to controls and FC between left thalamus and left caudate was correlated with disease severity (43). Spasmodic dysphonia (also called laryngeal dystonia) is a voice disorder that causes involuntary spasms of the larynx (44). As such, the FC abnormalities in regions associated with articulation in patients with spasmodic dysphonia mentioned above may represent specificity for abnormal muscle movements of the larynx. Thus, FC abnormalities in task specific focal dystonia appear to show some differentiation between focal dystonia type, particularly in cortical locations associated with the affected body part. In contrast to Parkinson's disease (neurodegenerative) who generally show *hypoconnectivity* within BG regions, patients with focal dystonias appear to often have *hyperconnectivity* within BG regions. Thus, MN FC in neonatal, pediatric, and adult pathology is associated with motor impairments and adult MN pathologies of Parkinson's and Alzheimer's disease show FC-disease differentiating attributes and widespread network effects.

There is also evidence that abnormal regional FC contributes to disruption of global FC in dystonia (45). However, another study determined that, while patients with focal dystonia had decreased functional connectivity regionally (within the striatum and between lateral primary sensorimotor cortex and the ventral intraparietal area), whole-brain global FC measures did not distinguish dystonia from HC after motion correction using global signal regression (46). In summary, focal dystonia in adults also show FC differences compared to controls, these differences appear to be primarily hyper connective within deep gray structures and often, but not always, hypoconnective in the cerebral cortex.

#### Functional Connectivity and Treatment of Movement Disorders

There have been several recent advances in knowledge about the potential utility of FC in predicting the effectiveness of deep brain stimulation (DBS) in Parkinson's disease, in particular, including the development of specialized toolboxes that provide pipelines for processing and integrating neuroimaging data for DBS (47, 48). Using a combination of normative healthy adult data and data from adults with Parkinson's disease, Horn et al. (49) demonstrated that anti-correlated FC between the subthalamic nucleus (DBS lead target) lead location and primary motor cortex based on normative or referenced FC data is predictive of clinical improvement after STN-DBS implantation. Preoperative FC between STN and the ipsilateral globus pallidus internus (GPi) has also been shown to be positively correlated with clinical improvement after STN-DBS in Parkinson's disease (50). In contrast, there is far less known about preoperative FC and DBS outcomes in patients with idiopathic generalized dystonia, however, a recent study positively correlated preoperative FC between lead site (GPi) and primary sensorimotor cortex, motor thalamus, and cerebellum (51). It should be noted that several of these connectivity studies also utilize structural connectivity (T2 diffusion imaging) although FC often independently predicts DBS outcomes (49, 51).

Finally, studies have also shown changes in FC to be associated with treatment for movement disorders. While this proves challenging for DBS (a pre-post FC design is technically difficult due to the challenges of MRI after DBS including both safety and susceptibility artifact introduced from DBS lead sites), there have been some observational studies investigating FC changes after treatment with botulinum toxin in focal (cervical) dystonia and blepharospasm (39, 40, 52–54). Taken together, these studies demonstrate that brain network changes associated with clinical treatment in movement disorders can be, respectively, measured with FC. For a summary of FC findings discussed, (see Table 1).

**Table 1:** Summary of functional connectivity findings reviewed by disease.

**Condition**	**Summary**
Parkinson's disease	FC in Parkinson's commonly follows a pattern of hypoconnectivity, particularly in BG but also often between BG and other regions. Methodologies focusing on the BG differentiate FC differences in Parkinson's compared to Alzheimer's disease. Methodologies not primarily focusing on BG may also see FC reductions associated with cortical-midbrain connections. Pre-op FC between STN and GPi is positively correlated with STN-DBS improvement. Normative (non-patient) FC between superimposed lead locations and M1 (anticorrelation) and SMA, anterior cingulate, and PFC (positive correlation) are associated with STN-DBS outcomes.
**Dystonia**	FC in dystonia seems to follow a pattern of almost always hyperconnectivity within/between deep-gray regions and then a mixture of hyper and hypo connectivity between deep gray and cortical/cerebellar regions as well as a mixture of hypo and hyperconnectivity between motor, sensory, association, and cognitive regions. At times, this is variable by study method. Hypoconnectivity between BG and cortical regions may also vary by type of dystonia (e.g., area affected by dystonia).
Generalized idiopathic dystonia	In patients, preoperative FC between GPi-DBS lead locations and M1/S1, motor thalamus, and cerebellum are positively correlated with outcomes, while FC between GPi-DBS lead locations and SMA and premotor cortex are negatively associated with outcomes.
Cervical dystonia	Often increased FC in BG and between BG and thalamus compared to controls. Some studies show hypoconnective FC between BG/thalamus and M1/S1 regions. Whole-brain studies show mixtures of increased and decreased FC between cortical regions. FC with regions of the cerebellum, within BG, and between BG and thalamus, M1, and S1 are reduced with botox injections. Some atypical (compared to HC) cortical FC is made normal or more HC-like after botox injections.
Blepharospasm/ Orofacial dystonia	Widespread reduced FC between caudate/putamen and cortical regions, as well as between cerebellar regions and between cerebellar and motor and visual regions compared to HC. After botox, widespread reduced FC including between BG and cerebellum, between BG structures, between cerebellar and visual and premotor (including frontal eye field) regions, between thalamus and premotor/SMA regions. Additionally, botox is associated with FC increases between cerebellum and associative visual cortex.
Writer's cramp	Increased BC FC (left putamen) and decreased FC within S1 and between motor hand area and superior parietal lobule. Additionally, FC in the intraparietal sulcus (associated with coordination of hand-eye movement) has been negatively corelated with disease duration and FC in parts of the superior parietal lobule associated with somatosensory guidance of movement were positively correlated with disease severity (Less FC with greater severity).
Musician's dystonia	Increased BG FC (right anterior putamen), these increases were not correlated with skill.
Spasmodic dysphonia	Increased FC between thalamus and BG as well as FC increases between many (cortical) sensorimotor, auditory, and cerebellar locations. Reduced FC between insula and semantic processing regions and well are between thalamus and motor (speech) regions. Connectivity between thalamus and caudate was positively associated with clinical severity.
Brain insult (perinatal-4 years)	Reduction of FC networks can be widespread and associated with motor outcomes, especially motor, sensory, BG, and cerebellar networks. Additionally, increased intra-hemispheric, decreased interhemispheric, and impaired lateralization of FC is associated with motor outcomes. However, very preterm infants had less association between BG networks and motor function scores and BG/thalamic networks were associated with posture).

*BG, basal ganglia; DBS, deep brain stimulation, FC, functional connectivity; GPi, globus pallidus internus; HC, healthy controls; M1, primary motor cortex; PFC, prefrontal cortex; Pre-op, preoperative; S1, primary somatosensory cortex; SMA, supplemental motor cortex; STN, subthalamic nucleus*.

## Advancements Through Effective Connectivity

FC demonstrates the potential of rs-fMRI signal to capture MN relevant characterizations both within and between regions of the brain and changes associated with age, ability, disease, and treatment. Although FC has revealed MN location and connectivity changes with age and disease, a different rs-fMRI connectivity approach, effective connectivity (EC), may be uniquely informative for clinically relevant dynamic MN-informed characterizations. EC is the causal influence that one node in a network (in this case, a neural system such as a brain region) over another (55) and generally is grounded in dynamic systems theory (56). Importantly, EC allows the user to not only infer functional connectivity between two regions (a descriptive relationship), but the ***direction*** of influence. That is, EC methods interrogate causal relationships of activation between brain regions (nodes) in order to find a reliable model for *how* data were caused specifically by addressing the temporal dynamics of the system [for wider review, see (55, 56)]. Given the complex neural circuitry involved in movement (such as the counterbalance of the direct and indirect pathways), this may prove beneficial for understanding not only why regions appear hyper- or hypo- connected but also the mechanistic changes of treatments.

Perhaps the most prevalent EC method using fMRI data is dynamic causal modeling [DCM - (57)]. DCM uses Bayesian model (comparison) framework to identify the model with the highest level of evidence. While there are multiple types of DCMs, in general, they specify a biophysically informed generative model including priors and use differential equations to describe hidden neuronal and physiological states based on observed data. This model space necessarily includes endogenous connections between nodes and may also include exogenous influences on nodes and/or connections (edges). In practice, at least two potential models are specified, inverted, and then their evidence is compared to determine which most likely explains the data. Methodologically, DCM in rs-fMRI, most performed using cross-spectral DCM (58) but also performed with stochastic DCM (59), is new compared to task-based (deterministic time series) fMRI DCM methods. In addition to DCM, common EC methods used include Granger causality analysis [GCA; (60, 61)] and Structural Equation Modeling [SEM; (7)]. In GCA, linear vector autoregressive models are fit to time-series data and compared per connection, often using likelihood ratio tests, using concepts of Granger causality. A more recent development of GCA is Granger causality mapping (GCM) which benefits from not requiring a priori specification of a model, thus allowing the user to take a more exploratory approach to identifying all EC for a given location (61, 62). SEM in functional neuroimaging makes inferences based on co-variances of neural activity among disparate regions within neuroanatomically-based models using a path analysis framework (63).

An important aside on EC methods: active task-based EC (active-EC) requires patient cooperation, whereas passive task-based EC (passive-EC) and purely resting state EC (rs-EC) have little to no patient cooperation demand, respectively, which has age-capacity success implications. For this review, active-EC refers to EC inferred from a volitional task requiring participation (e.g., finger tapping), passive-EC refers to EC inferred “epoch”-based periods during a scanning session but the difference during this period is not volitional on the part of the participant and they are instructed to be in a resting state (e.g., tremor-onset, activation of DBS). As studies of MN related rs-EC (or passive-EC) are relatively limited compared to FC, these sections will also review passive and active-EC studies.

### Motor Network Effective Connectivity in Healthy Adults

Healthy adults' active-EC is predictive of immediate future motor performance and downstream signaling network paradigms. For example, during adult response inhibition active-EC increases from the GPi to the thalamus, predicting both increased response inhibition and downstream active-EC from pre-SMA to right caudate (64). Such immediate behavioral and MN prediction capacity could hypothetically inform adjustment to deep brain stimulator (DBS) neuromodulation settings in disease. Also, this dynamic active-EC signaling paradigm is consistent with the indirect and hyperdirect model of BG signaling inhibition (64). These active-EC measures also correlated with the Unified Parkinson's Disease Rating Scale motor score and the finding that use of levodopa normalizes atypical FC patterns (64). Taken together, this suggests that active-EC measures have pathophysiologic meaning and could hypothetically accelerate DBS motor related improvements via informed stimulation parameter adjustments.

Active-EC in MN is also affected by implicit motor-sequence learning (finger tapping sequence) and shows different effects at the encoding stage [initial learning; (65, 66)] and after memory consolidation [repeating the task after sleeping; (66)]. Specifically, encoding has been associated with inhibitory effects from each M1 to the contralateral cerebellum (65) and inhibitory modulation from left M1 to right cerebellum, right premotor cortex to left cerebellum, and putamen to cerebellum bilaterally (66). In contrast, performance after memory consolidation was only associated with inhibition from left cerebellum to right putamen (66). Together, these studies suggest that both initial and more long-term stages of implicit, procedural, motor memory are associated with active-EC changes. This, again, has potential implications for identifying and guiding changes in treatments related to movements.

### Motor Network Effective Connectivity in Clinical Populations

Altered active-EC and passive-EC in adult BG circuitry has also been observed in movement disorders including Parkinson's disease passive-EC (67–69), and active-EC (68, 70) and focal dystonia active-EC (71, 72) (see Table 2 for summary of findings highlighted). For example, adults with writer's cramp dystonia compared to HC showed a mixture of abnormal excitatory and inhibitory active-EC within connections between motor cortex and the cerebellum, as well as BG, during a finger tapping task with the non-dominant hand (72). In a passive visual task, patients with cervical dystonia compared to HC showed greater “feedback” excitatory influence from bilateral striatum to the superior colliculus, a major hub for integrating sensory information (71). Thus, active-EC was able to localize the atypical connections during both active and passive tasks in patients with movement disorders. Additionally, in a study designed to identify whether DCM (active-EC) findings were reproducible in HC and if effects of Parkinson's disease and dopamine therapy were identifiable, Rowe et al. (70) found that a winning model was the same across two task sessions for HCs and also for medicated patients with Parkinson's, but the winning model was different for un-medicated Parkinson's patients. The authors state that winning model selection (circuit/model architecture) may be reliable enough to differentiate clinical populations, but do caution against relying on comparisons of parameter estimates (connection strengths) between groups (70). In addition to onset of actions, EC has been shown vary with symptoms. In a recent study by Dirkx et al. (69) in patients with Parkinson's, changes in Parkinsonian tremor amplitude (measured with EMG simultaneous to fMRI) were identified to have originated in the GPi which then drove changes throughout the cerebello-thalamo-cortico loop via first a GPi → M1 connection. Taken together, onset-based EC appears promising for identifying both onset of actions and symptoms.

**Table 2:** Summary of highlights of effective connectivity findings reviewed by disease.

**Condition**	**Summary**
Parkinson's disease	In passive-EC: DCM reliably differentiates HC from unmedicated Parkinson's patients (active-EC; 68). Passive-EC reliably identifies changes in BG directed connectivity from STN-DBS, including decreased strength of all connections to and from STN, and increased strength in connections in the direct pathway. The strength of connections in the direct and hyperdirect pathways are negatively correlated with clinical impairment and the degree of change in these pathways from STN-DBS is correlated with clinical efficacy of STN-DBS (67, 68). Additionally, the pathways modulated by STN-DBS are different at rest (hyperdirect and indirect) vs. during an active task (direct, indirect, hyperdirect, and CER → BG, M1 and CER self-connections) (68). Using passive-EC (STN-DBS stimulation): M1 and CER have bidirectional connectivity, but CER does not have EC connections with BG/thalamus (68). Using passive-EC (changes in tremor amplitude): Changes in tremor amplitude (simultaneous EMG-fMRI) are driven by GPi, these changes are propagated by a GPi → M1 connection through the cerebello-thalamo-cortico circuit and are ***not*** driven by an STN → CER connection (69).
Essential tremor	Using rs-EC pre and post thalamotomy: Transient increased self-inhibition of VL-thal, sustained effects of decreased excitation for SMA → DN, VL-thal → DN all compared to baseline. Additionally, increased Thal → DN excitation and reduced SMA → DN and VL-thal → DN excitation are associated with better posture scores, while only decreased SMA → DN is associated with better clinical motor action scores. In sum, transient changes are in the VL-thal self-connection, but sustained changes are in connections to DN. SMA → DN appears particularly important as it is also associated with clinical scores (73).
**Dystonia**	
Cervical dystonia	Using passive-EC (passive loom-recede visual stimulus): CD has greater Striatum → SC feedback connectivity than HC for looming stimulus, group differences were only seen when using a model that included “feedback” connections. (SC → striatum → thalamus → SC) (71).
Writer's cramp	Using active-EC during finger tapping task, WC show more excitation bidirectionally between M1 and CER and more inhibition from M1 → Put and Put → GPi. WC show less excitation bidirectionally between M1 and SMA, and between CER and Put; WC also show less GPi → M1 inhibition during task. This shows abnormalities in intracortical connections (hypo-excited), cortico-basal ganglia circuitry (both hyper and hypo excited), and cortico-cerebellar circuitry (hyper-excited) (72).
Spasmodic dysphonia	Using rs-EC: SD patients show stronger L IPC → L Putamen and stronger R to L PMC connections. SD with voice tremor also had stronger self-inhibition at locations compared to SD without voice tremor (74).

*CER, cerebellum; CD, cervical dystonia; DBS, deep brain stimulation DCM, dynamic causal modeling; DN, dentate nucleus (cerebellum); EC, effective connectivity; EMG, electromyography; HC, Healthy Control; GP, globus pallidus; GPi, globus pallidus internus; M1, primary motor cortex; PMC, premotor cortex; SC, superior colliculus; SD, spasmodic dysphonia; SMA, supplementary motor area; STN, subthalamic nucleus; Thal, thalamus; VL-thal, ventrolateral thalamic nucleus; WC, Writer's cramp*.

Differences in EC have also been noted with rs-EC. In adults with spasmodic dysphonia, both top-down (parieto-putaminal) and interhemispheric (right-to-left pre-motor) rs-EC is hyperexcitable and patients with and without tremor can be further differentiated by the fact that patients with tremor additionally show less self-inhibition for the left parietal cortex, left putamen, and right premotor cortex (74). Thus, rs-EC was able to localize atypical directional connectivity in focal dystonia as well as differentiate sub-groups of similar patient groups.

Importantly, passive-EC and rs-EC have demonstrated potential for localizing and characterizing pathological dynamic network signal on an individual basis to guide effective surgical therapies in adults, such as the positioning of modulatory intracerebral leads or ablations (67, 73). Such proof of concept is exemplified in Parkinson's disease, where passive-EC by dynamic causal modeling (DCM) has been used to model the effects of subthalamic nucleus (STN)-DBS on BG circuitry, and changes of directional modulation induced with “on” STN-DBS are associated with clinical efficacy of DBS (67). Specifically, they found that the endogenous strength of the connections within the direct (putamen → thalamus) and hyperdirect pathways (M1 → STN) were negatively correlated with clinical impairment and modulation of these pathways from STN-DBS was positively correlated with clinical improvement from STN-DBS. Additionally, the strength of the connection putamen → STN was positively correlated with clinical impairment. Importantly, the primary modulatory effects of STN-DBS were reproduced in subsequent study using the same paradigm in a different cohort of patients (68). Reproducing these effects, although at the same institution and with the same group, speaks to a strength of this paradigm and also highlights the importance of considering methodological differences when results to different studies seem paradoxical. This is of particular importance when considering clinical groups and the potential to inform biomarkers/diagnosis and treatment.

One additional advantage of passive-EC and rs-EC paradigms for investigating the effects of DBS is that directional computational modeling techniques, such as DCM, can model “hidden” nodes in a network. This is beneficial because, post DBS implant, an MRI susceptibility artifact will occur at lead locations, thus making it not possible to measure the BOLD response at lead location. However, as techniques such as DCM are able to model lead-inferred location activity (LILA) within a network of regions by placing a “hidden” node at the lead location, it can use rs-fMRI to infer the effect of DBS on brain-network dynamics (59, 67, 68, 75). Crucially, while using this technique (67, 68) identified several changes centering around the “hidden” STN node, suggesting that it correctly identified STN as a location pivotal to clinical changes despite having not directly measured the STN. Similarly, rs-EC in essential tremor, pre and post-thalamotomy, also predicts post-operative dynamic MN changes and clinical motor-symptom scores without the use of “hidden nodes,” and some clinical correlations included regions affected by surgery (73). This suggests that rs-EC also characterizes casual network dynamics with high enough integrity to guide surgical strategy and is associated with clinical outcomes in adults with essential tremor. Specifically, rs-EC allows for the identification of regions that are functionally connected, as well as the direction and nature of those connections. Certain techniques also allow for LILA, opening possibilities for inferring neural activity at lead sites and identifying causal network dynamic changes (not restricted to lead sites) as a function of DBS. As such, rs-EC may lead to higher specificity and precision in identifying underlying network neuropathology.

## Limitations

While rs-EC (and passive-EC) is a promising method for investigating and developing clinically relevant and actionable biomarkers, it is important to note the limitations and methodological considerations. fMRI itself faces limitations. A recent paper called into question the ability for fMRI techniques and analyses to adequately control for false-positives, reporting a potential false positive rate of up to 70% for clusterwise inferences if inadequate corrections are performed (76). This is particularly relevant if whole-brain activation analysis is used to identify potential regions of interest for model nodes in EC and for FC whole-brain analyses. Care must be taken to use appropriate analysis methods and multiple comparisons corrections in whole-brain analyses (particularly those that use familywise error) when using activation-based ROIs. Additionally, it is critical to note the importance of adequately denoising rs-fMRI data, especially in the context of movement (46, 77, 78). Movement is a prevalent source of noise in rs-fMRI and the risk of movement is generally higher in children and clinical populations. Finally, in terms of methodological considerations, it is important to note that reproducibility of findings can be influenced by differences in data-acquisition, pre-processing, analysis paradigm, ROI selection, and more.

## Conclusion

Functional connectivity builds upon the work established by structural modalities in characterizing MN configuration, is associated with lifespan MN related changes, has pathology-localizing potential, has potential to localize FC related changes after medical treatments including medications and botulinum injection, and relates to motor behavior in health and disease across neonatal to adult age groups. However, effective connectivity may prove to be particularly useful in brain-invasive (DBS) MN-informed therapies due to its potential to demonstrate lead-inferred location activity, especially as DBS systems have begun to be compatible with 3T MRI. Passive-EC and rs-EC potential may hold promise in childhood movement disorder therapies, given the lower to no patient demand during a resting state fMRI scan.

## Impact Statement

While functional connectivity has elucidated much MN properties with relation to age, disease, and behavior, effective connectivity has been shown to be useful in MN-informed therapies in adults. Thus, effective connectivity may have potential to impact childhood movement disorder therapies, given the lower to no patient demand.

## Author Contributions

BS, SW, VB, and MK: drafting the work. BS, VB, MK, SW, AW, JH, MG, and PDA: conception and design of article, revising for intellectual content, final approval of version to be published, and agreement to be accountable for all aspects of the work in ensuring that questions related to the accuracy or integrity of any part of the work are appropriately investigated and resolved. All authors contributed to the article and approved the submitted version.

## Conflict of Interest

The authors declare that the research was conducted in the absence of any commercial or financial relationships that could be construed as a potential conflict of interest.

## Publisher's Note

All claims expressed in this article are solely those of the authors and do not necessarily represent those of their affiliated organizations, or those of the publisher, the editors and the reviewers. Any product that may be evaluated in this article, or claim that may be made by its manufacturer, is not guaranteed or endorsed by the publisher.
